# Viral Infection Increases Glucocorticoid-Induced Interleukin-10 Production through ERK-Mediated Phosphorylation of the Glucocorticoid Receptor in Dendritic Cells: Potential Clinical Implications

**DOI:** 10.1371/journal.pone.0063587

**Published:** 2013-05-08

**Authors:** Sinnie Sin Man Ng, Andrew Li, George N. Pavlakis, Keiko Ozato, Tomoshige Kino

**Affiliations:** 1 Unit on Molecular Hormone Action, Program in Reproductive and Adult Endocrinology, Eunice Kennedy Shriver National Institutes of Child Health and Human Development, National Institutes of Health, Bethesda, Maryland, United States of America; 2 School of Biomedical Science, Faculty of Medicine, The Chinese University of Hong Kong, Shatin, Hong Kong Special Administrative Region, China; 3 Human Retrovirus Pathogenesis Section, Vaccine Branch, Center for Cancer Research, National Cancer Institute, Frederick, Maryland, United States of America; 4 Laboratory of Molecular Growth and Regulation, Eunice Kennedy Shriver National Institutes of Child Health and Human Development, National Institutes of Health, Bethesda, Maryland, United States of America; McMaster University, Canada

## Abstract

The hypothalamic-pituitary-adrenal axis plays a central role in the adaptive response to stress including infection of pathogens through glucocorticoids. Physical and/or mental stress alter susceptibility to viral infection possibly by affecting this regulatory system, thus we explored potential cellular targets and mechanisms that underlie this phenomenon in key immune components dendritic cells (DCs). Dexamethasone (DEX) treatment and subsequent Newcastle disease virus (NDV) infection most significantly and cooperatively stimulated mRNA expression of the interleukin (IL)-10 in murine bone marrow-derived DCs among 89 genes involved in the Toll-like receptor signaling pathways. NDV increased DEX-induced IL-10 mRNA and protein expression by 7- and 3-fold, respectively, which was observed from 3 hours after infection. Conventional DCs (cDCs), but not plasmacytoid DCs (pDCs) were major sources of IL-10 in bone marrow-derived DCs treated with DEX and/or infected with NDV. Murine cytomegalovirus and DEX increased serum IL-10 cooperatively in female mice. Pre-treatment of DCs with the extracellular signal-regulated kinase (ERK) inhibitor U0126 abolished cooperative induction of IL-10 by DEX and NDV. Further, ERK overexpression increased IL-10 promoter activity stimulated by wild-type human GR but not by its mutant defective in serine 203, whereas ERK knockdown abolished NDV/DEX cooperation on IL-10 mRNA and phosphorylation of the mouse GR at serine 213. NDV also increased DEX-induced mRNA expression of three known glucocorticoid-responsive genes unrelated to the Toll-like receptor signaling pathways in DCs. These results indicate that virus and glucocorticoids cooperatively increase production of anti-inflammatory cytokine IL-10 by potentiating the transcriptional activity of GR in DCs, through which virus appears to facilitate its own propagation in infected hosts. The results may further underlie in part known exacerbation of IL-10/T helper-2-related allergic disorders by stress and viral infection.

## Introduction

Glucocorticoids (GCs), end products of the hypothalamic-pituitary-adrenal (HPA) axis, play a central role in the body’s adaptive response to changes in external and internal environment, called “stressors” [1]. Infection of pathogens, such as of viruses and bacteria, is one of such external stressors, potently activating the HPA axis and inducing subsequent secretion of GCs from the adrenal cortex. Pathogens stimulate central part of this regulatory system (e.g., brain hypothalamus and pituitary corticotrophs) directly with their structural and genetic components, and indirectly with cytokines and inflammatory mediators secreted from activated immune cells and infected tissues [2]. Secreted GCs in turn subside inflammation, functioning as a counter regulatory mechanism for otherwise overshooting immune response [3]. GCs do this by suppressing cellular immunity and production of T-helper (Th) 1 cytokines, such as the interleukin (IL)-12, tumor necrosis factor (TNF) α and the interferon (IFN) γ, while they stimulate humoral immunity and secretion of Th2-related anti-inflammatory cytokines, including IL-4, IL-10 and the transforming growth factor β [4], [5]. Since GCs have diverse and strong immunosuppressive effects, they are widely used as potent therapeutic compounds in the treatment of allergic, autoimmune and inflammatory diseases, acute sepsis and shock, and organ rejection [6]. At the molecular level, most of the known anti-inflammatory actions of GCs are mediated by the glucocorticoid receptor (GR), a member of the nuclear hormone receptor superfamily [7]. Actions of the GR are under strict regulation by other signaling pathways with various modes of actions, including phosphorylation of its N-terminal domain by several serine/threonine kinases [8].

Dendritic cells (DCs) play a pivotal and critical role in the anti-pathogen immunity [9]. They detect infected pathogens via their pattern recognition receptors like the Toll-like receptors (TLRs) [10]. Upon sensing pathogens, these cells activate naive T-cells in lymphoid organs with their co-stimulatory molecules expressed on their cell surface, such as CD80, CD86 and CD40 [11], [12], as well as by producing tremendous amounts of type I IFNs (IFNα and β) and several pro-inflammatory cytokines, including IL-6, IL-12 and TNFα, ultimately stimulating the overall inflammatory reaction against pathogens and infected tissues [11]. Interestingly, DCs also secrete the anti-inflammatory cytokine IL-10 in response to pathogen infection [13]. Indeed, DCs are the major sources of IL-10, secreting this cytokine particularly in the late phase of their immune response (∼24 hours after infection), in contrast to the pro-inflammatory cytokines whose secretion they start from a relatively early period (few hours after infection) [13]–[15]. These characteristic profiles of cytokine secretion by DCs indicate that they organize not only activation of pro-inflammatory reaction, but also resolution of inflammation.

It has long been reported that activation of the HPA axis by mental and/or physical stress and subsequent elevation of circulating GCs increases susceptibility to infectious diseases, prolong and/or worsen their disease course [16]. We thus hypothesized that pathogens could increase anti-inflammatory actions of GCs to facilitate their infection and invasion to tissues. We focused on DCs, central components of the immune response against pathogen infection, and found that viral infection potently enhanced GC-induced expression of IL-10 and other glucocorticoid-responsive genes by phosphorylating GR through activation of ERK.

## Materials and Methods

### Mice

Male and female C57BL/6 mice (6–8 week old) were used as sources for bone marrow-derived DCs and for the infection study described below. This study was carried out under the regulation of the Guide for the Care and Use of Laboratory Animals of the National Institutes of Health. The Animal Study Proposal, which covered experiments described in the manuscript was approved in advance by the NICHD Animal Care and Use Committee with the proposal number: 09-008. Mice were housed in the NICHD animal facility. Injection of dexamethasone (DEX) and the murine cytomegalovirus performed through peritoneal cavity did not cause distress in animals. At the end-point of experiments, mice were euthanized with carbon dioxide gas and their respiration was carefully followed up.

### Generation of Bone Marrow-derived DCs

Mouse bone marrow-derived DCs were generated by culturing bone marrow cells in the presence of 100 ng/ml of the FMS-like tyrosine kinase 3 ligand (FLT3L) for 8 days, as described previously [17]. We employed FLT3L-induced DCs in this study, as they represent steady-state DCs resident in lymphoid organs, in contrast to the granulocyte/macrophage-colony-stimulating factor- or IL-4-induced DCs, which are equivalents of the DCs that emerge after inflammation [18]. After 4 days of culture, half of the medium was removed and replenished with fresh medium supplemented with FLT3L (100 ng/ml), and the cells were incubated for an additional 4 days to finally develop into bone marrow-derived DCs. Over 98% of these cells expressed CD11c, indicating that the bone marrow precursors cells differentiated into DCs. For separation of pDCs, FLT3L-induced DCs were incubated with B220 microbeads (Miltenyi Biotech, Cambridge, MA). pDCs were captured and sorted by AutoMACS (Miltenyi Biotech) according to the manufacturer’s instructions, while flow-through cells were used as cDCs.

### Infection/stimulation of DCs

DCs (1×10^6^ cells) were pre-treated with 10^−6^, 10^−7^ or 10^−8 ^M of dexamethasone (DEX) (Sigma Aldrich St. Louis, MO) or 10^−6^ M of corticosterone (CORT) (Sigma Aldrich, St. Louis, MO) for 30 min. In some experiments, DCs were also incubated 30 min prior to addition of DEX with the GR inhibitor RU486 (10^−5^ M), ERK inhibitor U0126 (0.1 M to 10 M), p38 MAPK inhibitors SB203580 (10 µM) and MT4 (10 µM) or the protein kinase A (PKA) inhibitor H-89 (12.5 nM). All these compounds were purchased from EMD Biochemicals Inc. (Darmstadt, Germany). Steroid/compound-treated cells were then infected with 10 multiplicity of infection (MOI) units of the Newcastle disease virus (NDV) (Hertz strain) or murine cytomegalovirus (MCMV) (Smith strain) purchased from American Type Culture Collection (Manassas, VA). Viability of DCs after these treatments was verified by trypan blue staining and was always over 98%.

### RNA Isolation and PCR Array

Total RNA was isolated from DCs, and was treated with DNase I by using the RNeasy Mini Kit (Qiagen, Valencia, CA) according to the protocol of the manufacturer. RT^2^ Profile TLR Signaling PCR Array (SA Bioscience, Frederick, MD) was used to examine simultaneously the mRNA levels of 89 genes associated with TLR signaling pathways and five housekeeping genes [β-glucuronidase (*GUSB*), hypoxanthine guanine phosphoribosyl transferase 1 (*HPRT1*), heat shock protein 1 (*HSPCB*), glyceraldehyde 3-phosphate dehydrogenase *(GAPDH)* and β-actin (*ACTB*)] in 96-well plates using the ABI Prism 7500 Real-time PCR System (Applied Biosystems, Foster City, CA) according to the protocol of the manufacturer. Each reaction included the cDNA converted from 380 ng of total RNA. Obtained threshold cycle (C_t_) values were normalized by mean C_t_ values of the five housekeeping genes, and fold changes were calculated by using the comparative C_t_ method (2^−ΔΔCt^ where ΔΔC_t_ = ΔC_t,sample_−ΔC_t,reference_). Three mice were used for each treatment group.

### Real-time Quantitative PCR (qPCR)

Total RNA (0.5 µg) obtained at indicated time points were converted to cDNA using the TaqMan reverse transcription reagents and oligo-dT as a primer (Applied Biosystems). qPCR was performed with 20 ng of cDNA, 5 µM of primers and 12.5 µl of the SYBR green PCR Master Mix (Applied Biosystems) in a total volume of 25 µl. All qPCRs were conducted in triplicate. Sequences of the primers used in qPCR were listed in Table S1. C_t_ values of the examined molecules were normalized with those of GAPDH, and fold changes were obtained by using the comparative C_t_ method.

### IL-10 Enzyme-linked Immunosorbent Assay (ELISA)

Protein concentrations of IL-10 were determined in culture media used for incubation of DCs (1×10^6^ cells) or sera obtained from mice by using the murine IL-10 ELISA kit (R&D Systems Inc., Minneapolis, MN).

### In vivo MCMV Infection Study

Female mice were injected intraperitoneally twice (24 hours and 1 hour prior to viral infection) with the veterinary injection grade DEX (0.3 µg/g animal) (NIH pharmacy, Bethesda, MD). They were then injected intraperitoneally with 1×10^6^ plaque formation unit (pfu) of MCMV. The experiment was performed in triplicate and was repeated twice. Mice were sacrificed 48 hours after MCMV injection, and their sera and livers were collected.

### Histological Analysis

Livers obtained from the female mice with indicated treatments were fixed, stained with hematoxylin & eosin (H&E), and were analyzed microscopically. Total numbers of inflammatory sites defined as discrete clusters of inflammatory cells were counted in entire sections, and their representative images were recorded (10× and 40× magnifications).

### Transfection and Luciferase Reporter Assay

HCT116 cells and RAW264.7 cells were cultured respectively in McCoy’s 5A medium or Dulbecco’s modified Eagle’s medium supplemented with 10% FCS and antibiotics (Invitrogen, Carlsbad, CA). They were transfected for 6 hours by using Lipofectamine 2000 (Invitrogen) with 0.2 µg/ml of the plasmid expressing wild type human GR, or a mutant human GR harboring S203A replacement [19], 0.2 µg/ml of the plasmid expressing wild type mouse ERK1 or ERK2 (Addgene, Cambridge, MA), or a constitutively active ERK2 mutant harboring D319N replacement (Addgene), together with 0.5 µg/ml of the pGL3-based luciferase reporter plasmid containing the full length murine IL-10 promoter (Gift from Dr. M. Tone, Cedars-Sinai Medical Center, Los Angeles, CA) [20] and 0.5 µg/ml of the pGL4.73[*hRluc*/SV40] renilla control plasmid (Promega, Madison, MI). pGILZ-luc, which contains the glucocorticoid-responsive human glucocorticoid-induced leucine zipper (GILZ) promoter, was also used in some experiments [21]. Some RAW264.7 cells were transfected with 1 µg of siRNAs for mouse ERK1 and ERK2 (Santa Cruz Biotechnology Inc., Santa Cruz, CA). Cells were then treated with DEX (10^−6^ M) for an additional 24 hours. Lysates were analyzed for the firefly and renilla luciferase activities by using the Dual-luciferase Assay Kit and the GloMax Luminometer (Promega).

### Western Blot

DCs (1×10^7^ cells) were infected with NDV (MOI = 10) and/or treated with DEX (10^−6^ M), and the whole cell extracts were prepared by using protein extraction kit (Active Motif, Carlsbad, CA). Whole cell extracts (5 µg) were run on 8–12% NuPAGE Bis-Tris gels (Invitrogen), transferred to nitrocellulose membranes and were immunoblotted with the anti-total ERK1/2, anti-phospho ERK1/2 (at Thr202/Tyr204), anti-phospho GR (at serine 203 [human] and 213 [mouse]) (Cell Signaling Technology, Danver, MA), anti-GR or anti-β-actin antibody (Santa Cruz Biotechnology Inc.).

### Statistical Analysis

Statistical analyses were performed with Student’s t test with two-tailed p value using the Prism 5 software (GraphPad Inc., La Jolla, CA). p-Values less than 0.05 were considered as statistically significant.

## Results

### DEX Pre-treatment Modulates the Expression of Molecules Associated with TLR Signaling upon Viral Infection in DCs

**Figure 1:**
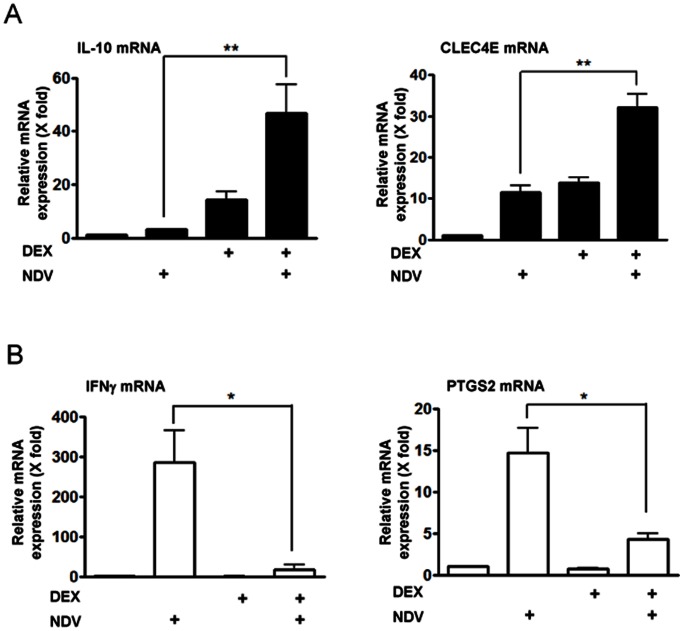
DEX pre-treatment and NDV infection cooperatively regulated mRNA expression of the four genes in DCs. Bone marrow-derived DCs were pre-treated with DEX (10^−6 ^M) for 30 min, and were infected with NDV (MOI = 10) for 6 hours. mRNA levels of top four genes in Table S2 were measured in the real-time qPCR by using newly designed primers (panel **A**: *IL-10* and *CLEC4E*: genes whose mRNA expression was cooperatively enhanced by DEX pre-treatment and NDV infection, panel **B**: *PTGS2* and *IFNγ* : genes whose mRNA expression was stimulated by NDV infection but repressed by DEX pre-treatment). Their C_t_ values were normalized with those of *GAPDH*, and relative mRNA expression (fold changes) was calculated by comparing to the control obtained in the absence of DEX pre-treatment and NDV infection. Bars represent means and standard errors of fold changes of their mRNA expression obtained in three independent experiments. *: p<0.05, **: p<0.01, compared the 2 conditions indicated.

To study the effect of GC treatment and viral infection on DC-mediated immune response, we first pre-treated FLT3L-induced bone marrow-derived DCs with DEX, infected with NDV, and quantitatively measured mRNA expression of 89 genes whose products play important roles in the TLR signaling pathways and anti-pathogen response promoted by these cells. Genes examined included those encoding the TLRs (TLR1 to 10), TLR adaptor proteins (such as MyD88 and Tollip), downstream signal intermediate molecules (such as Irak1 and TAK1), end effector molecules and the transcription factors (c-Jun N-terminal kinases (JNK), p38 mitogen-activated protein kinase (MAPK) and components of the nuclear factor of κB and the interferon regulatory factors (IRFs), and their target molecules (IL-1β, IL-6, IL-10, IFNβ and CXCL10). Among the genes significantly regulated by DEX pre-treatment and/or NDV infection (Table S2), genes encoding IL-10 and the C type-lectin domain family 4 member e (CLEC4E) demonstrated cooperative induction of their mRNAs by DEX pre-treatment and NDV infection, while those for IFNγ, prostaglandin-endoperoxide synthase 2 (PTGS2) and IFNβ1 demonstrated significant increase of their mRNA expression upon NDV infection, which was further suppressed by DEX pre-treatment. We verified mRNA expression of 4 most significantly regulated genes by using qPCRs with newly designed primers (Figure 1 and Table S1). As expected, NDV enhanced DEX pre-treatment-induced mRNA expression of *IL-10* and *CLEC4E* in DCs (Figure 1A), while DEX pre-treatment suppressed NDV-induced mRNA expression of *PTGS2* and *IFNγ* (Figure 1B).

*PTGS2* expresses the cyclooxygenase-2, which is a rate-limiting enzyme for synthesizing prostaglandins, bioactive lipids essential for maturation, migration and inflammatory response of DCs [22]–[24]. IL-10 is an anti-inflammatory cytokine that suppresses viral infection-induced tissue inflammation by affecting various components of the immune system [25], [26]. Indeed, DCs are major sources of circulating IL-10 secreted in response to viral infection, while they are also prime targets of this cytokine, being influenced in an autocrine or paracrine fashion [27]. In contrast, majority of IFNγ is produced by natural killer cells and T-cells, thus this cytokine secreted from DCs contributed minimally to its entire activity [26], [28]. Because of a critical and major role of IL-10 in DC-mediated anti-inflammatory response, we further evaluated details of the cooperative induction of IL-10 by DEX and NDV, and explored underlying mechanism(s).

### DEX- and NDV-induced Cooperative Induction of IL-10 in DCs: Time Course, Protein Expression and Involvement of GR

**Figure 2:**
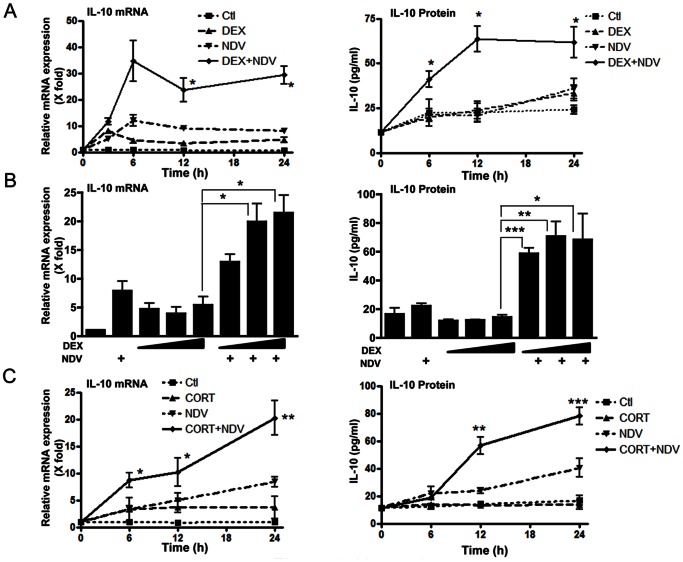
DEX pre-treatment and NDV infection cooperatively increased IL-10 mRNA and protein expression in DCs. DCs were pre-treated with DEX (**A**: 10^−6^ M, **B**: 10^−6^, 10^−7^ or 10^−8 ^M) or CORT (**C**: 10^−6^ M) for 30 min, and were infected with NDV (MOI = 10) for 6 hours. Time-course of IL-10 mRNA (**A**, left panel) and protein (**A**, right panel) expression, and the effect of increasing concentrations of DEX on these parameters (**B**, left and right panel, respectively) are shown. Time-courses of IL-10 mRNA (left panel) and protein (right panel) expression obtained in the presence of CORT pre-treatment are also shown in panel **C**. Relative IL-10 mRNA expression (fold changes) was calculated by comparing to the baseline (the conditions at time “0″ or those obtained in the absence of DEX/CORT pre-treatment and NDV infection). Bars represent means and standard errors of fold changes of the IL-10 mRNA expression and protein concentrations in the culture media obtained from three independent experiments. *: p<0.05, **: p<0.01, ***: p<0.001, compared to the conditions obtained in the presence of DEX or CORT treatment alone. Ctl: control.

**Figure 3:**
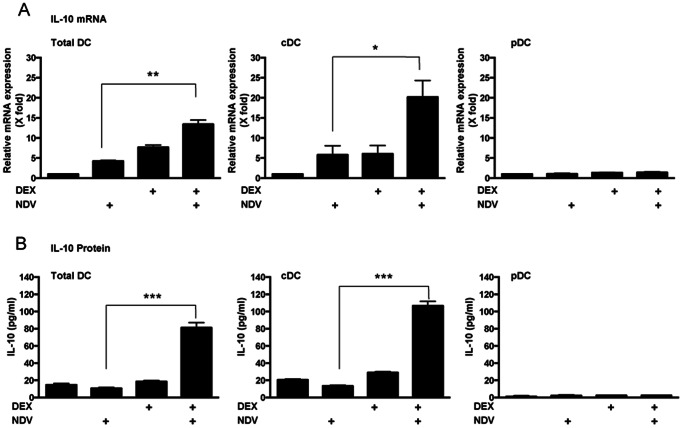
cDCs, but not pDCs, are the major DC subtype for production of IL-10 upon DEX treatment and/or NDV infection in the FLT3L-derived DCs. FLT3L-derived DCs were sorted into cDC and pDC subpopulation. They were individually pre-treated with DEX (10^−6 ^M) for 30 min, infected with NDV for 24 hours, and IL-10 mRNA expression (**A**) and IL-10 protein expression (**B**) were measured. Bars represent means and standard errors of fold changes of the IL-10 mRNA expression and protein concentrations in the culture media obtained from three independent experiments. *: p<0.05, **: p<0.01, ***: p<0.001, compared the 2 conditions indicated.

We first examined time course of the DEX- and NDV-induced cooperative effect on the mRNA expression of IL-10 in DCs. This effect was observed from 3 hours after NDV infection and sustained for up to 24 hours (Figure 2A, left panel). We next examined time course of the IL-10 protein secreted into culture media by using the mouse IL-10-specific ELISA. DEX and NDV cooperatively increased IL-10 production at all time points after infection (Figure 2A, right panel). Their peak cooperation was observed at 12 hours after viral infection with 3-fold increase. NDV increased the effect of all tested concentrations of DEX (10^−6^ to 10^−8 ^M) on IL-10 mRNA and protein expression (Figure 2B). CORT, a major endogenous form of GCs in mouse, also induced IL-10 mRNA and protein expression by cooperating with NDV, although the observed effect was weaker than that by DEX, possibly due to its less potency compared to the latter synthetic glucocorticoid (Figure 2C) [29]. We sorted FLT3L-induced DCs into the two major DC subtypes, cDCs (CD11c+/B220-) and pDCs (CD11c+/B220+) (Figure S1), and individually treated them with DEX and/or infected with NDV. We observed robust induction of IL-10 in cDCs but not in pDCs (Figure 3A & B), suggesting that the major DC subtype that produces IL-10 in our experimental system is cDCs, This result is consistent with the previous reports that pDCs do not produce IL-10 upon virus infection or TLR stimulation [18], [30], [31].

**Figure 4:**
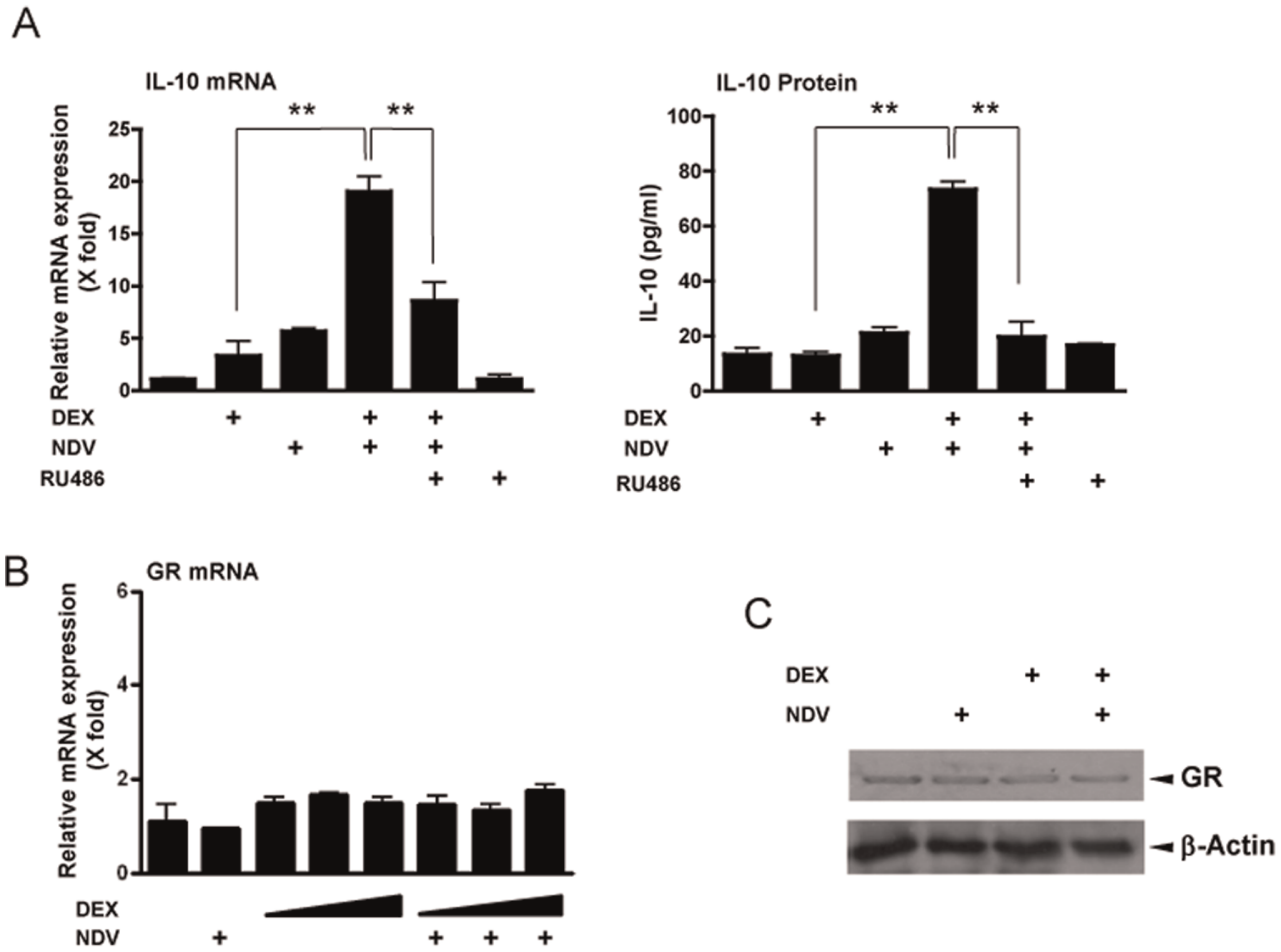
GR mediated DEX pre-treatment- and NDV infection-induced cooperative IL-10 mRNA expression in DCs. **A**. RU486 suppressed DEX-induced increase of IL-10 mRNA and protein expression in DCs. DCs were pre-treated with RU486 (10^−5^ M) and/or DEX (10^−6^ M) for 30 min, and were infected with NDV (MOI = 10) for 6 hours. IL-10 mRNA and protein expression are shown. Bars represent means and standard errors of fold changes of the IL-10 mRNA expression and protein concentrations in culture media obtained from three independent experiments. **: p<0.01, compared to the 2 conditions indicated. **B & C**. DEX pre-treatment and NDV infection did not change GR mRNA and protein expression in DCs. DCs were pre-treated with DEX (10^−6^ M) for 30 min, and were infected with NDV (MOI = 10) for 6 hours. Bars in panel **A** and **B** represent means and standard errors of fold changes of the mRNA expression obtained in three independent experiments. Whole cell extracts obtained from DCs were run on SDS-PAGE gels, and GR and control β-actin were visualized with their specific antibodies in Western blots in panel **C**.

To examine if GR is required for cooperative induction of IL-10 by GCs and NDV, we pre-treated DCs with the receptor antagonist RU486 prior to DEX treatment [32]. RU486 attenuated NDV- and DEX-induced IL-10 mRNA and protein expression (Figure 4A). DEX treatment and/or NDV infection did not alter GR mRNA and protein expression (Figure 4B and 4C). These results indicate that the observed effect of DEX pretreatment and NDV infection on IL-10 expression was mediated by GR, and not by altering expression of this receptor.

### DEX Pre-treatment and MCMV Infection Cooperatively Increase IL-10 Production in Mice and Reduce Inflammation in their Livers

**Figure 5:**
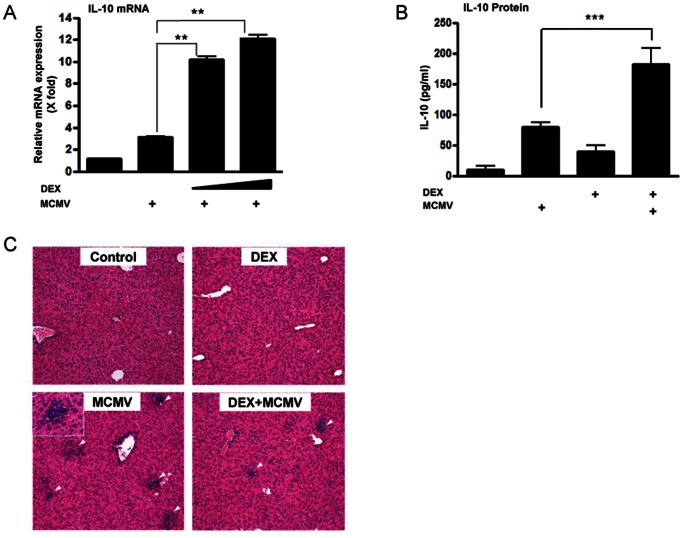
DEX pre-treatment and MCMV infection cooperatively induced IL-10 production *in vitro* and *in vivo*, and suppressed inflammation in the liver. **A**. DEX pre-treatment and MCMV infection cooperatively increased IL-10 mRNA expression in DCs. DCs were pre-treated with DEX (10^−7^ or 10^−8^ M) for 30 min, and were infected with MCMV (1×10^6^ pfu) for 6 hours. Bars represent means and standard errors of fold changes of the IL-10 mRNA expression. **: p<0.01, compared to the 2 conditions indicated. **B & C**. DEX pre-treatment and MCMV infection cooperatively induced IL-10 production in mice and suppressed inflammation in their livers. Female mice were pre-treated with DEX (0.3 µg/g animal) twice (at 24 hours and 1 hour prior to viral injection), and were infected with MCMV (1×10^6^ pfu). Two days after the infection, sera and livers were collected. Panel **B** shows alternation in the serum IL-10 concentrations in mice, while panel **C** demonstrates representative images of the H&E staining of their livers (magnification 10×). The inset of the left lower panel shows an inflammatory site in the liver caused by MCMV infection (magnification 40×). Bars represent means and standard errors of the serum IL-10 concentrations obtained from three mice. ***: p<0.001, compared to virus infection alone.

We examined whether the cooperation of GC pre-treatment and viral infection on IL-10 expression could also be observed at animal levels. For this purpose, we employed MCMV as an infecting agent, as infection of this virus enhanced DEX-induced IL-10 mRNA expression similar to NDV in DCs (Figure 5A) [33]. In addition, MCMV has more restricted host specificity than NDV, thus it is a much safer pathogen for animal studies, potentially diminishing a chance of infection to other animals closely housed in the same animal facility. DEX treatment and MCMV infection respectively increased serum levels of IL-10 by ∼10- and ∼5-fold, while their simultaneous treatment further increased serum concentrations of IL-10 (Figure 5B). As expected, DEX pre-treatment significantly decreased numbers of the inflammatory sites in the liver (Figure 5C & Table S3), indicating that DEX efficiently suppressed host immune response against virus-infected tissues. Taken together, these *in vivo* results suggest that GCs and viral infection cooperatively increase IL-10 production, which may in part contribute to suppressing the host immune response against tissues and organs infected by MCMV.

### ERK Plays a Key Role in DEX- and NDV-induced Cooperative IL-10 Expression in DCs

**Figure 6:**
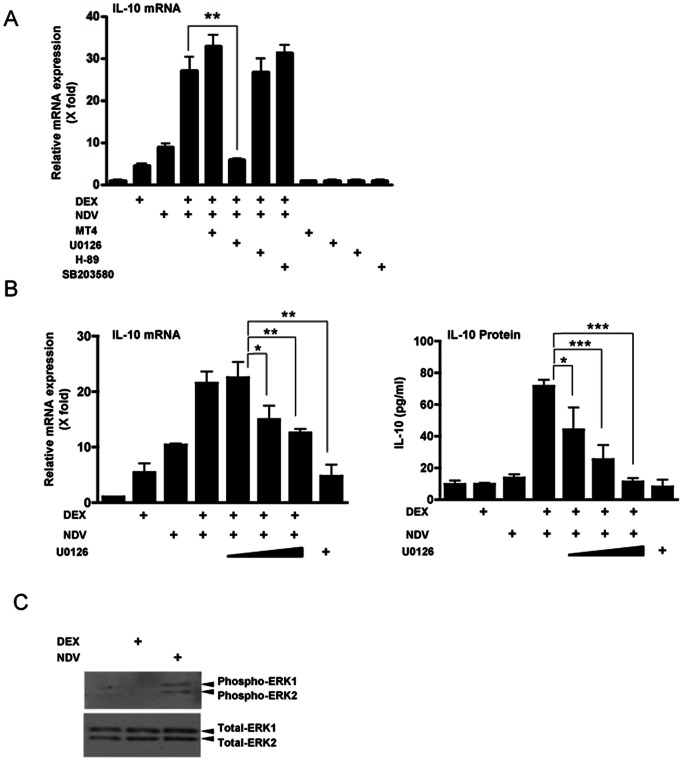
ERK1/2 mediated DEX- and NDV-induced cooperative IL-10 mRNA expression in DCs. **A.** ERK inhibitor U0126 abolished DEX- and NDV-induced cooperative IL-10 mRNA expression in DCs. DCs were pre-treated with indicated kinase inhibitors for 30 min (p38 MAPK inhibitors SB203850 (1 µM) and MT4 (10 µM); the ERK inhibitor U0126 (0.1 M); and the PKA inhibitor H-89 (12.5 nM)). They were then treated with DEX (10^−6 ^M) for 30 min, and were infected with NDV (MOI = 10) for 6 hours. **: p<0.01, compared the 2 conditions indicated. **B.** ERK inhibitor U0126 dose-dependently reduced DEX-and NDV-induced cooperative IL-10 mRNA and protein expression in DCs. DCs were pre-treated with increasing concentrations of U0126 (0.1 M, 1 M and 10 M), and were treated with DEX (10^−6 ^M) for 30 min. They were then infected with NDV (MOI = 10) for 6 hours. Bars represent means and standard errors of fold changes of the IL-10 mRNA (left panel) and protein concentrations in culture media (right panel). *: p<0.05, **: p<0.01, ***: p<0.001, compared to the 2 conditions indicated. **C.** NDV infection but not DEX pre-treatment activated ERK1/2 in DCs. DCs were treated with DEX (10^−6 ^M), or were infected with NDV (MOI = 10) for 6 hours. Whole cell extracts were obtained after 24 hours of infection, and were run on SDS-PAGE gels. Phosphorylated ERK1/2 and their entire fraction were visualized with their specific antibodies in Western blots.

For stimulating production of IL-10, DCs employ several distinct kinases, such as p38 and ERK MAPKs and PKA, to mediate virus-activated TLR signaling toward downstream effector molecules [27], [34]–[36]. Thus, we examined the effect of their inhibitors on DEX- and NDV-induced stimulation of IL-10 expression in DCs. The ERK inhibitor U0126 significantly attenuated the cooperative effect of DEX and NDV on IL-10 mRNA expression in DCs, while the p38 MAPK inhibitors SB203580 and MT4, and the PKA inhibitor H-89 failed to do so (Figure 6A). We also found that U0126 suppressed DEX- and NDV-induced IL-10 mRNA and protein expression in a dose-dependent fashion (Figure 6B). We confirmed that NDV infection induced phosphorylation of ERK1/2, a marker for the activation of these kinases, while DEX pre-treatment did not show obvious effects (Figure 6C). These results suggest that ERK activation is required for NDV to increase DEX-induced IL-10 expression in DCs.

### NDV Cooperatively Stimulates IL-10 Expression with DEX by Phosphorylating GR through ERK

**Figure 7:**
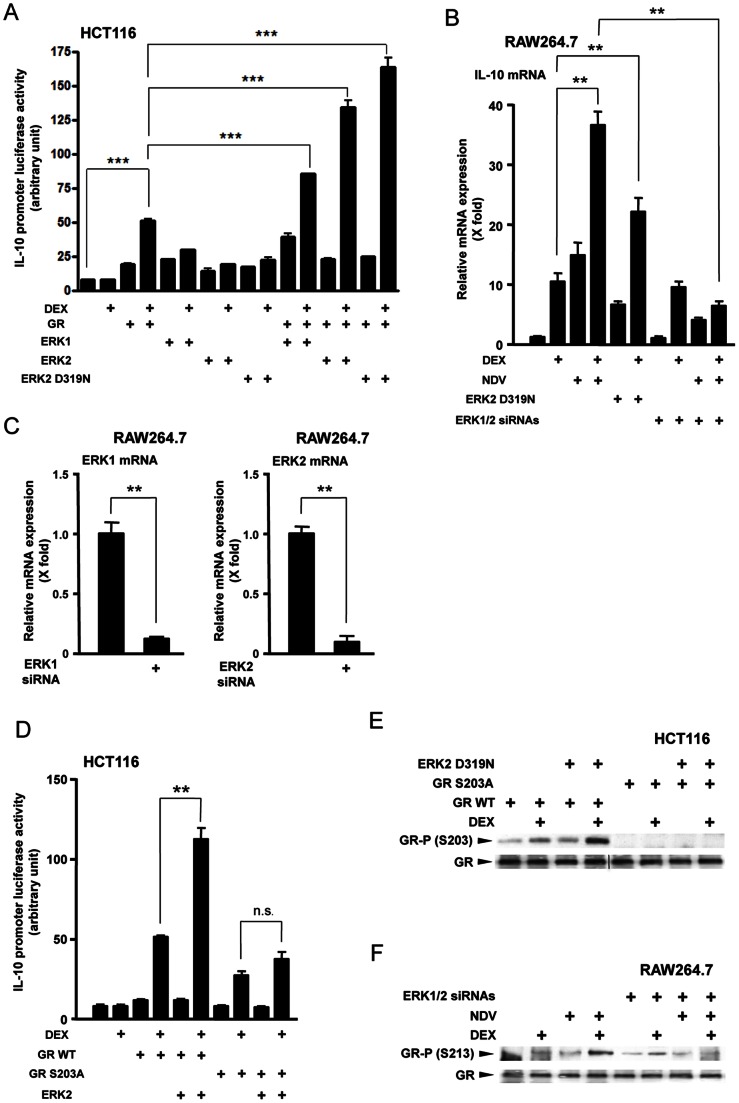
NDV infection increased IL-10 expression by phosphorylating GR at serine 203 (human) and 213 (mouse). **A**. ERK increased GR-induced transcriptional activity of the IL-10 promoter in HCT116 cells. HCT116 cells were transfected with wild type ERK1-, 2- or its constitutively active mutant ERK2 D319N-expressing plasmid in the presence of wild type human GR, together with the pGL3 reporter carrying the IL-10 promoter and the pGL4.73[*hRluc*/SV40] control plasmid. Cells were subsequently treated with DEX (10^−6^ M) for 24 hours. Bars represent means and standard errors of the firefly luciferase activity corrected for renilla luciferase activity. ***: p<0.001, compared the 2 conditions indicated. **B & C**. NDV infection increased DEX-induced IL-10 mRNA expression though ERK1/2 in RAW264.7 cells. RAW264.7 cells were transfected with ERK2 D319N-expressing plasmids and/or ERK1/2 siRNAs and treated/infected with DEX (10^−6^ M) and/or NDV for 24 hours. The effect of ERK1/2 siRNA on the mRNA expression of ERK1 and 2 was shown in panel **C**. Bars represent means and standard errors of fold changes of the IL-10, ERK1 and ERK2 mRNA. **: p<0.01, compared the 2 conditions indicated. **D**. ERK2 increased DEX-induced IL-10 promoter activity in the presence of wild type human GR but not in the presence of its mutant defective in serine 203 in HCT116 cells. HCT116 cells were transfected with wild type ERK2-expressing plasmid in the presence of wild type human GR or its mutant with S203A replacement, together with the pGL3 reporter carrying the IL-10 promoter and the pGL4.73[*hRluc*/SV40] control plasmid. Cells were subsequently treated with DEX (10^−6^ M) for 24 hours. Bars represent means and standard errors of the firefly luciferase activity corrected for renilla luciferase activity. **: p<0.01, n.s.: not significant, compared the 2 conditions indicated. **E**. ERK2 D319N increased DEX-induced phosphorylation of wild type human GR at serine 203, while it had no effect on the mutant GR with S203A replacement in HCT116 cells. HCT116 cells were transfected with ERK2 D319N-expressing plasmid together with wild type GR- or GR S203A mutant-expressing plasmid, and were treated with DEX (10^−6^ M) for 30 min. Whole cell extracts obtained from these cells were run on SDS-PAGE gels and the GR phosphorylated at serine 203 and its entire fraction were visualized with their specific antibodies in Western blots. **F**. NDV infection increased DEX-induced phosphorylation of mouse GR at serine 213, while ERK1/2 knockdown abolished the NDV effect in RAW264.7 cells. RAW264.7 cells were transfected with ERK1/2 siRNAs and treated/infected with DEX (10^−6^ M) and/or NDV for 30 min. Total cell lysates obtained from these cells were run on SDS-PAGE gels and the mouse GR phosphorylated at serine 213 and its entire fraction were visualized with their specific antibodies in Western blots.

To further examine role(s) of ERKs on the cooperative induction of IL-10 by NDV and DEX, we overexpressed ERK1, ERK2 or a constitutively active mutant ERK2 D319N in HCT116 cells, and examined their effects on DEX-stimulated GR transcriptional activity on the IL-10 promoter in reporter assays. Although HCT116 cells are not immune cells, they are useful for examining the activity of GR mutants defective in phosphorylation sites, because they do not express endogenous GR [19]. We found that DEX treatment increased IL-10 promoter activity by 10-fold in the presence of wild type GR, consistent with previous results [37]. Both ERK1 and ERK2 further increased DEX-stimulated IL-10 promoter activity with the latter demonstrating a more pronounced effect (Figure 7A). A constitutively active mutant ERK2 D319N showed the strongest enhancement on the DEX-stimulated promoter activity [38]. Both NDV infection and transfection of the ERK2 D319N-expressing plasmid enhanced DEX-induced IL-10 mRNA expression in mouse monocytoid RAW264.7 cells, while co-transfection of ERK1/2 siRNAs abolished the effect of NDV infection on DEX-induced IL-10 mRNA expression (Figure 7B). ERK1/2 siRNAs reduced the mRNA expression of *ERK1* and *ERK2* by 90% (Figure 7C). It is known that several serine/threonine kinases phosphorylate GR at serine residues located in its N-terminal domain (such as those at amino acid position 203, 211 and 226 of the human GR, and 213, 220 and 235 of the mouse GR), and modulate GR-induced transcriptional activity [19], [39]. We therefore expressed mutant human GRs defective in these serines together with ERK2, and found that replacement of serine 203 with alanine completely abolished ERK2-mediated enhancement of DEX-stimulated IL-10 promoter activity (Figure 7D). In contrast, ERK2 was still active on the transcriptional activity of the mutant human GRs defective in serine 211 or 226 (data not shown). We further found that ERK2 D319N enhanced DEX-induced phosphorylation of wild type human GR, but not of the mutant GR defective in serine 203 in HCT116 cells (Figure 7E). NDV infection also enhanced GR phosphorylation in a DEX treatment-dependent fashion in RAW264.7 cells, while transfection of ERK1/2 siRNAs abolished NDV-induced phosphorylation of mouse GR at serine 213 (Figure 7F). These results indicate that the ERKs activated by NDV infection, and subsequent phosphorylation of GR by these kinases [at serine 203 (human) and 213 (mouse)] mediate the cooperative stimulation of IL-10 expression by NDV and DEX.

### NDV and DEX Cooperatively Stimulate mRNA Expression of 3 Known Glucocorticoid-responsive Genes Unrelated to the Toll-like Receptor Signaling Pathways through Phosphorylation of GR

**Figure 8:**
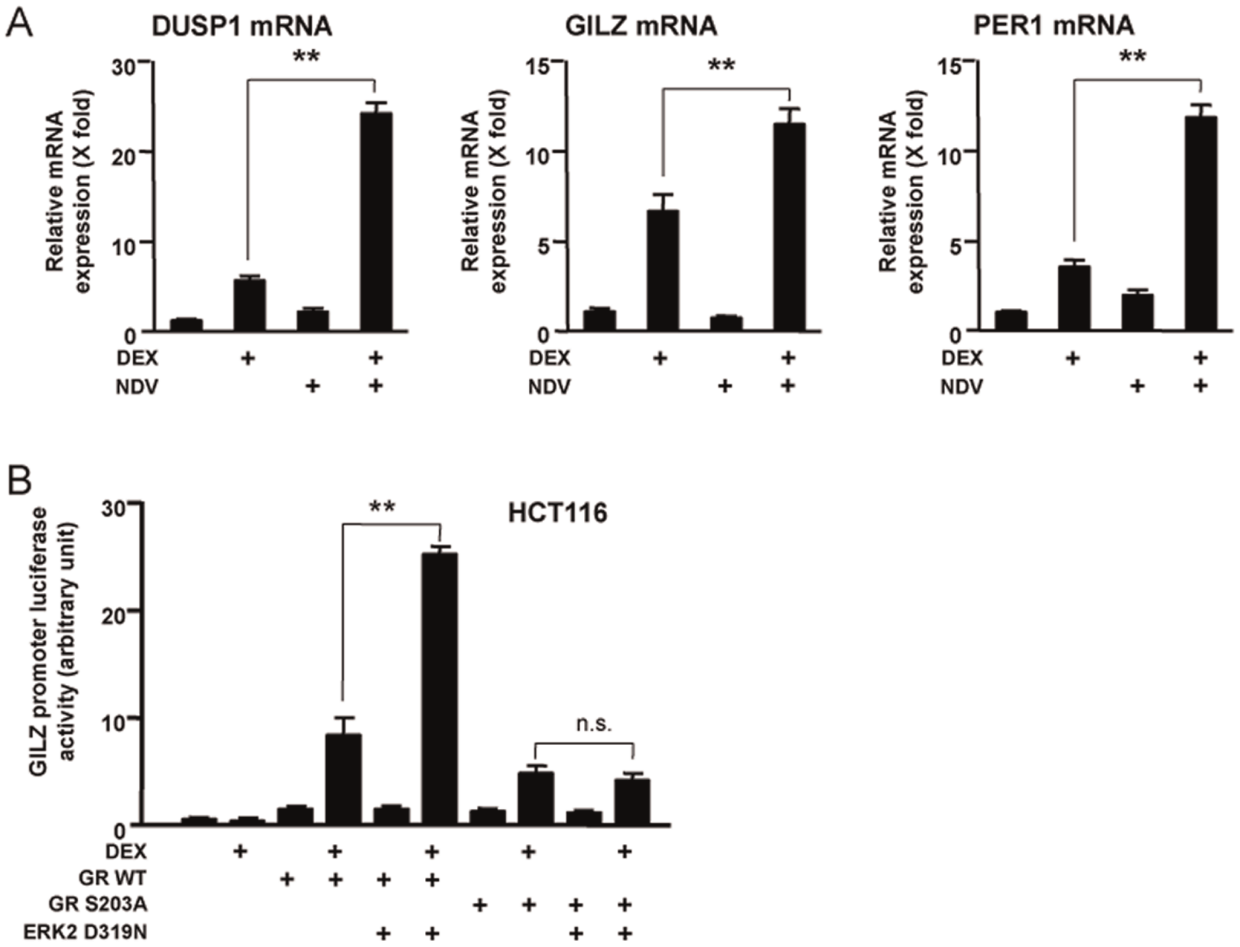
NDV increases DEX-induced expression of several glucocorticoid-responsive genes through phosphorylation of GR. **A**. NDV increased DEX-induced mRNA expression of 3 well-known glucocorticoid-responsive genes in DCs. DCs were pre-treated with DEX (10^−6 ^M) for 30 min, and were infected with NDV (MOI = 10) for 6 hours. mRNA levels of *DUSP1*, *GILZ* and *PER1* were measured in the real-time qPCR. Bars represent means and standard errors of fold changes of their mRNA expression obtained in three independent experiments. **: p<0.01, compared the 2 conditions indicated. **B**: ERK2 increased DEX-induced transcriptional activity of the GILZ promoter in the presence of wild type human GR in HCT116 cells, while it lost the effect in the presence of the mutant GR harboring S203A replacement. HCT116 cells were transfected with ERK2 D319N-expressing plasmid in the presence of wild type human GR or its mutant with S203A replacement, together with the pGILZ-luc reporter and the pGL4.73[*hRluc*/SV40] control plasmid. Cells were subsequently treated with DEX (10^−6^ M) for 24 hours. Bars represent means and standard errors of the firefly luciferase activity corrected for renilla luciferase activity. **: p<0.01, n.s.: not significant, compared the 2 conditions indicated.

ERK appears to phosphorylate GR regardless of its tethering to the IL-10 promoter, and thus, is expected to modulate GR transcriptional activity on other glucocorticoid-responsive genes. We therefore examined mRNA expression of 3 well-known glucocorticoid-responsive genes unrelated to the TLR signaling pathways in DCs, which were treated with DEX and infected with NDV (Figure 8A). DEX stimulated mRNA expression of the dual-specificity phosphatase 1 (*DUSP1*), *GILZ* and the period 1 (*PER1*) by over 5-fold and NDV infection strongly increased mRNA expression of all of these genes in a DEX-dependent fashion. NDV however minimally influenced their basal mRNA expression. To verify contribution of ERK and its phosphorylation of GR to the observed cooperative induction of their mRNA expression by NDV and DEX, we employed *GILZ* as a representative gene. DEX stimulated the GILZ promoter activity by ∼10-fold in the presence of wild type human GR in HCT116 cells, and overexpression of ERK2 D319N strongly enhanced DEX-stimulated GR transcriptional activity on this promoter (Figure 8B). ERK2 D319N lost its enhancing effect on the transcriptional activity of the mutant human GR harboring serine to alanine replacement at amino acid position 203. Taken together, these results suggest that NDV infection generally modulates the expression of glucocorticoid-responsive genes through activation of ERK and subsequent phosphorylation of GR in DCs.

## Discussion

In this study, we evaluated the cooperative effect of GCs and NDV in mouse bone marrow-derived DCs on the expression of 89 genes whose products play important roles in the TLR signaling pathways. Among the genes differentially regulated by DEX and/or NDV, we focused on *IL-10*, as mRNA expression of this gene showed the most significant change. In addition, DCs are the major immune cells that secrete this cytokine [26]. NDV infection enhanced DEX pre-treatment-induced IL-10 mRNA expression and protein production in DCs. This effect of NDV started as early as 3 hours after infection and persisted for 24 hours. MCMV and DEX cooperatively enhanced production of IL-10 in mice. NDV activated ERK1/2, phosphorylated human GR at serine 203 (213 in mice) and ultimately increased the transcriptional activity of GR, not only on the *IL-10* gene but also on other TLR-unrelated glucocorticoid-responsive genes.

IL-10 is an anti-inflammatory cytokine, which suppresses inflammation by affecting functions of various immune cells [26]. Upon viral infection, circulating levels of IL-10 increase after ∼24 hours to 20 days in mice to facilitate resolution of inflammation promoted by pro-inflammatory cytokines, which are secreted into circulation immediately (∼3–6 hours) after infection [13], [15]. Therefore, IL-10 acts as a negative regulatory factor against the pro-inflammatory immune response, which is beneficial for clearing pathogens from infected organisms but whose excessive and prolonged activation is detrimental to local inflammatory tissues [14]. Thus, the levels and duration of IL-10 production are tightly regulated during the course of immune response against pathogens, while dysregulation in this process may result in prolonged/persistent infection and even systemic anergy to infected organisms [26], [40]. We found that cooperation between DEX and NDV on the IL-10 production started as early as 3 hours after the viral infection in DCs. Thus, it is quite possible that some viruses distort normal regulation of IL-10 secretion in these cells through cooperation with GCs, and increase their propagation in host tissues. Indeed, several viruses in the *Herpesviridae* family, such as the Epstein-Barr virus and the varicella-zoster virus, encode IL-10-like molecules, which share immunosuppressive properties of host IL-10, and increase their infectivity to and latency in their hosts [41]. MCMV, which we found to induce IL-10 production cooperatively with DEX, is also a member of this family [42], suggesting that IL-10 and its downstream biologic actions are common targets for some members of this viral family to modulate host immune activity. Further, persistent infection of *Mycobacterium tuberculosis* and reactivation of its previous inflammatory sites are also associated with excessive production of IL-10 [40]. As we found in the original screening, DEX pre-treatment and NDV infection cooperatively altered mRNA expression of several genes including *CLEC4E*, *IFNγ*, *PTGS2* and *IFNβ1*, in addition to *IL-10*. Thus, it is possible that virus also modulates host immune response by changing expression of these genes through cooperation with GCs.

Our results on the cooperation of DEX and virus on IL-10 production may in part explain the previous observation that mental/physical stress increases susceptibility to viral infection and tendency to exacerbate/prolong its disease course [16]. This hypothesis may be supported by our results that the NDV-induced enhancement of IL-10 expression was particularly observed with the strong synthetic glucocorticoid DEX as well as with high concentrations of CORT frequently encountered in stressed animals. Further, our results may also provide a potential mechanistic explanation to the exacerbation of bronchial asthma by physical/emotional stress and viral infection, as elevation of IL-10 production and resulting activation of Th-2-directed humoral immunity play important pathogenetic roles in this potentially lethal airway disease [43]–[48].

We demonstrated that NDV infection enhanced GC-stimulated production of IL-10 by phosphorylating GR through ERK in DCs. It is known that several viruses activate ERK in macrophages, alveolar epithelial A549 cells and primary tracheobronchial epithelial cells [49]–[51]. Since IL-10 promoter does not contain GREs [52], ERK appears to enhance indirect transcriptional activity of GR on this promoter. In preliminary experiments, we observed that addition of some TLR ligands (TLR7/8 and TLR9) increased IL-10 mRNA levels in DCs similar to viral infection (data not shown), thus it is possible that NDV infection activated ERK pathway through stimulation of the specific pattern recognition receptor(s), such as TLR7/8 and 9. In addition to these TLRs, RNA viruses activate p38 MAPK through the cytoplasmic helicase RIG-like receptors [53]. Poly(I:C), a synthetic double stranded RNA, stimulates several MAPKs including ERK in neutrophils, although involvement of RIG-like receptors in the activation of ERKs have not been verified as yet [54]. These pieces of evidence may suggest that RIG-like receptors also play a role in the activation of ERK in response to infection of RNA viruses in DCs.

Some serine/threonine kinases including p38 MAPK, JNK and the cyclin-dependent kinases (CDKs), phosphorylate several serine residues located in the N-terminal domain of GR and positively or negatively modulate the transcriptional activity of this receptor [19], [39]. Activation of GR by ligand is necessary for ERK to phosphorylate this receptor possibly due to their ligand-dependent interaction, although virus activates this kinase independently to glucocorticoids. As reported in the case of CDK5 and p38 MAPK, phosphorylation-mediated alteration in the transcriptional cofactor attraction to the activation function-1 domain of GR may be one of the mechanisms underlying the ERK-mediated enhancement of GR transcriptional activity [19], [55]. We found that NDV-induced GR phosphorylation enhanced DEX-induced mRNA expression of several well-known glucocorticoid responsive, GRE-containing genes in addition to IL-10. This result suggests that NDV can potentially modulate expression of many glucocorticoid-responsive genes in addition to this cytokine by phosphorylating GR. Indeed, NDV may also modulate through phosphorylation of GR the expression of other TLR signaling pathway-related genes, which we found to be regulated by DEX and NDV in our screening. It is reported that the respiratory syncytial virus (RSV), which is one of the major causes of lower respiratory tract infection and hospital visits during infancy and childhood, represses the anti-inflammatory action of glucocorticoids through GR [56]. Since RSV is in the same *Paramyxoviridae* family as NDV, it is possible that RSV modulates the anti-inflammatory action of these hormones by phosphorylating GR through activation of ERK.

In conclusion, we described a novel cooperation between viral infection and GCs on the expression of glucocorticoid-responsive genes in DCs through phosphorylation of GR by ERK. Through this activity particularly on IL-10, viruses may increase their propagation in host organisms by suppressing the latters’ immune activity.

## Supporting Information

Supplemental Figure 1 **Composition of cDC and pDC in Flt3L-derived DCs.**
(PDF)

Supplemental Table 1 **List of the primers used in qPCR.**
(PDF)

Supplemental Table 2 **TLR signaling-associated molecules whose mRNA expressions were modulated by DEX pre-treatment and NDV infection in DCs.**
(PDF)

Supplemental Table 3 **The effect of MCMV infection and dexamethasone treatment on the formation of focal inflammatory sites in the liver.**
(PDF)
