# Supplementary figures and images for: Viral Infection Increases Glucocorticoid-Induced Interleukin-10 Production through ERK-Mediated Phosphorylation of the Glucocorticoid Receptor in Dendritic Cells: Potential Clinical Implications
Source: PLoS One. 2013 May 8;8(5):e63587. doi: 10.1371/journal.pone.0063587 (PMC3648469; doi:10.1371/journal.pone.0063587)

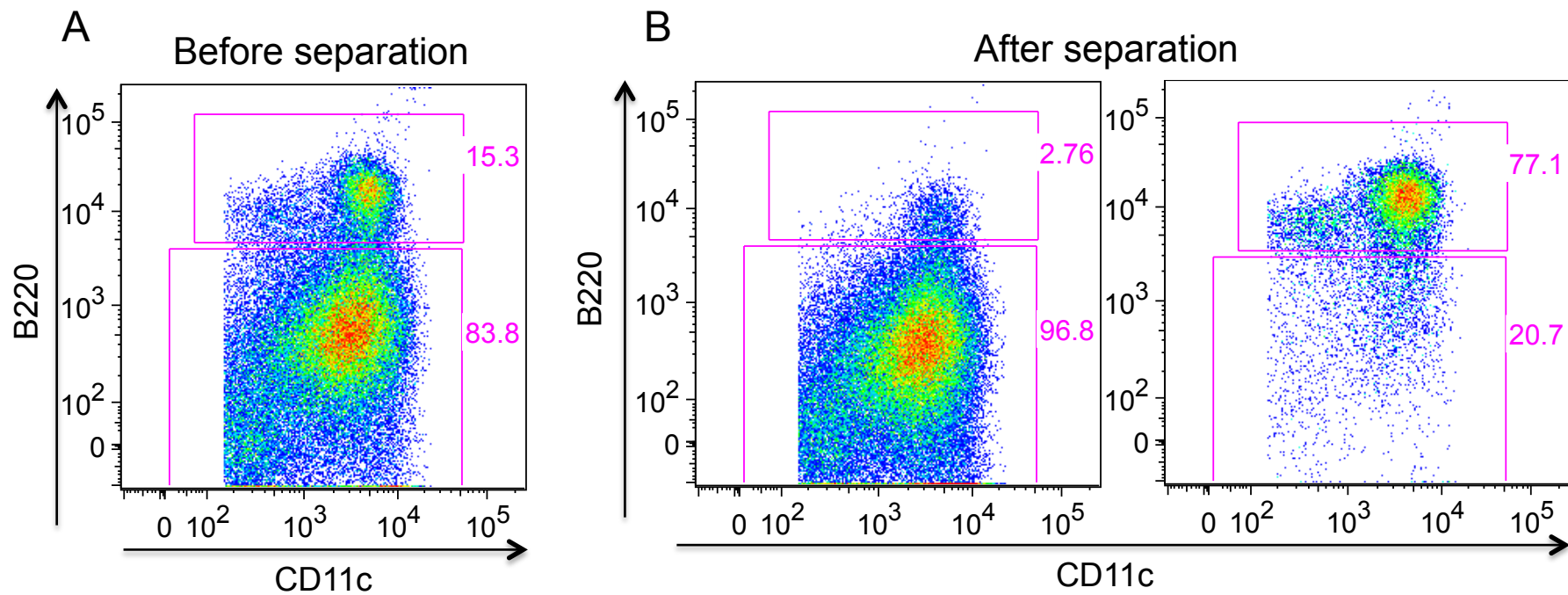

Supplemental figure 1, Ng *et al.*

Supplement: Supplemental Figure 1 — Composition of cDC and pDC in Flt3L-derived DCs. (PDF) [file pone.0063587.s001.pdf]
