# Supplementary material for: Viral Infection Increases Glucocorticoid-Induced Interleukin-10 Production through ERK-Mediated Phosphorylation of the Glucocorticoid Receptor in Dendritic Cells: Potential Clinical Implications
Source: PLoS One. 2013 May 8;8(5):e63587. doi: 10.1371/journal.pone.0063587 (PMC3648469; doi:10.1371/journal.pone.0063587)
Supplement: Supplemental Table 1 — List of the primers used in qPCR. (PDF) [file pone.0063587.s002.pdf]

Supplemental Table 1. List of the primers used in qPCR.

Gene	Symbol	Primer sequence
C-type lectin domain family 4, member e	*CLEC4E*	Forward: 5'-TGGGGGCTCACCTGGTGGTT-3'<br>Reverse: 5'-CCATTGCCACTGACCCTCCACC-3'
Extracellular signal-regulated kinase 1	*ERK1*	Forward: 5'-CACGCAGCTGCAGTACATC-3'<br>Reverse: 5'-CAGCAAGATCTGGATCTC-3'
Extracellular signal-regulated kinase 2	*ERK2*	Forward: 5'-CTACGGCATGGTTTGC-3'<br>Reverse: 5'-GATGTTCTCATGTCTGAAG-3'
Dual-specificity phosphatase 1	*DUSP1*	Forward: 5'-GATCGCCGACCTCAAGAAATGG-3'<br>Reverse: 5'-CAAGGCGTCAAGCATATCC-3'
Glucocorticoid-induced leucine zipper	*GILZ*	Forward: 5'-GACCATGCTCTCCATTCTAC-3'<br>Reverse: 5'-CACGAATCTGCTCCTTTAG-3'
Glucocorticoid receptor	*GR*	Forward: 5'-GTTCCTAAGGAAGGTCTGAAGAG-3'<br>Reverse: 5'-CAATTCTGACTGGAGTTTCC-3'
Glyceraldehyde 3-phosphate dehydrogenase	*GAPDH*	Forward: 5'-GTGTTCCTACCCCCAATGT-3'<br>Reverse: 5'-TGTCATCATACTTGGCAGGTTTC-3'
Interferon γ	*IFNγ*	Forward: 5'-GAGGAACTGGCAAAAGGATG-3'<br>Reverse: 5'-GCTGATGGCCTGATTGTCTT-3'
Interleukin-10	*IL-10*	Forward: 5'-CCTGGTAGAAGTGATGCCCC-3'<br>Reverse: 5'-TCCTTGATTTCTGGGCCATG-3'
Period 1	*PER1*	Forward: 5'-CTGTGTCAAGCAGGTTCAG-3'<br>Reverse: 5'-GAAGGTGTCCTGGTTTCG-3'
Prostaglandin-endoperoxide synthase 2	*PTGS2*	Forward: 5'-AGGGCCCTTCCTCCCGTAGC-3'<br>Reverse: 5'-TGAGCCTTGGGGGTCAGGGA-3'
