# Supplementary material for: Viral Infection Increases Glucocorticoid-Induced Interleukin-10 Production through ERK-Mediated Phosphorylation of the Glucocorticoid Receptor in Dendritic Cells: Potential Clinical Implications
Source: PLoS One. 2013 May 8;8(5):e63587. doi: 10.1371/journal.pone.0063587 (PMC3648469; doi:10.1371/journal.pone.0063587)
Supplement: Supplemental Table 2 — TLR signaling-associated molecules whose mRNA expressions were modulated by DEX pre-treatment and NDV infection in DCs. (PDF) [file pone.0063587.s003.pdf]

Supplemental Table 2. TLR signaling-associated molecules whose mRNA expression was modulated by DEX pre-treatment and NDV infection in DCs.

Genes	Symbol	Treatment		
		DEX	NDV	DEX+NDV
Interleukin 10	*IL-10*	10±1.19 *	5.1±0.56 *	76.3±15.13 *
C-type lectin domain family 4 member e	*CLEC4E*	14.9±0.13*	15.0±3.33*	41.4±11.00*
Interferon γ	*IFNγ*	1.9±2.53	1453.7±114.73 *	77.4±6.23 *
Prostaglandin-endoperoxide synthase 2	*PTGS2*	-0.5±0.76	20.4±2.86 *	4.9±0.35 *
Interferon β1	*IFNβ1*	2.3±0.56	55873.1±0.23*	37702.9±4.79*
Chemokine (C-X-C motif) ligand 10	*CXCL10*	-16.4±6.5*	1336.9±90.09*	1421.8±133.55*
Lymphotoxin A	*LTA*	-3.2±1.24	545.5±149.07*	288.174.39*
Heat shock protein 1A	*HSPA1A*	1.5±0.29	213.0±30.53*	124.5±23.72*
Interleukin 6	*IL-6*	-0.5±1.03	114.4±24.42*	52.3±5.23*
Interleukin 1a	*IL-1a*	-0.2±0.93	21.3±12.02*	14.0±8.23*
Chemokine (C-C motif) ligand 2	*CCL2*	-0.7±0.38	19.6±4.67*	15.3±2.99*
CD80 antigen	*CD80*	-1.1±0.06	19.4±1.05*	12.1±1.34*
Tumor necrosis factor	*TNF*	-2.2±0.41	18.4±3.25*	12.7±1.75*
CD86 antigen	*CD86*	0.4±0.72	18.0±1.89*	16.3±2.03*
Eukaryotic translation initiation factor 2a kinase 2	*EIF2AK2*	-1.6±0.11*	10.4±1.21*	10.0±1.27*
Tumor necrosis factor a induced protein 3	*TNFAIP3*	-0.5±0.79	9.8±1.21*	11.4±1.39*
Interferon regulatory factor 1	*IRF1*	-1.9±0.16*	8.4±0.27*	7.0±1.21*
Pellino 1	*PELI1*	-1.4±0.02*	7.6±0.90*	8.1±0.72*
Nuclear factor of k light chain gene enhancer in B-cells inhibitor, α	*NFKG1α*	3.2±0.45*	6.1±0.49*	8.2±0.99*
Receptor (TNFRSF)-interacting serine-threonine kinase 2	*RIPK2*	-0.4±0.71	5.8±1.23*	6.0±0.55*
Reticuloendotheliosis oncogene	*REL*	-1.7±0.12*	5.8±1.21*	3.6±0.67*
TANK-binding kinase 1	*TBK1*	-1.6±0.07*	5.4±0.97*	5.8±0.74*
Interleukin 6 receptor subunit α	*IL-6Rα*	2.1±0.08*	-8.4±0.09*	-3.30.44*
ELK1, member of ETS oncogene family	*ELK1*	-0.6±0.96	-5.0±1.40*	-4.2±0.11*
Interleukin-1 receptor-associated kinase 1	*IRAK1*	-2.1±0.29*	-6.2±0.16*	-4.9±0.69*
Interleukin 1β	*IL-1β*	-84.4±0.45 *	1.7±0.23 *	-14.4±4.79 *

fold change >5

fold change <-5

*: $p < 0.05$, compared to the baseline obtained in the absence of DEX and NDV treatment.
