# Supplementary material for: Viral Infection Increases Glucocorticoid-Induced Interleukin-10 Production through ERK-Mediated Phosphorylation of the Glucocorticoid Receptor in Dendritic Cells: Potential Clinical Implications
Source: PLoS One. 2013 May 8;8(5):e63587. doi: 10.1371/journal.pone.0063587 (PMC3648469; doi:10.1371/journal.pone.0063587)
Supplement: Supplemental Table 3 — The effect of MCMV infection and dexamethasone treatment on the formation of focal inflammatory sites in the liver. (PDF) [file pone.0063587.s004.pdf]

Supplemental Table 3. The effect of MCMV infection and dexamethasone treatment on the formation of focal inflammatory sites in the liver.

Treatment	Number of inflammatory sites (/0.1 m^2 liver)	
	**Mean**	**S.E.**
Control	0.20	0.21
DEX	0.33	0.23
MCMV	133.20	14.41
DEX + MCMV	79.40*	9.59

*: p=0.036 (n=3), compared to MCMV treatment.
